# Triiodothyronine Potentiates BMP9-Induced Osteogenesis in Mesenchymal Stem Cells Through the Activation of AMPK/p38 Signaling

**DOI:** 10.3389/fcell.2020.00725

**Published:** 2020-07-31

**Authors:** Xiaoting Chen, Yan Hu, Tianyuan Jiang, Chao Xia, Yan Wang, Yanhong Gao

**Affiliations:** Department of Geriatrics, Xinhua Hospital, Shanghai Jiao Tong University School of Medicine, Shanghai, China

**Keywords:** Bone morphogenetic protein 9, triiodothyronine, osteogenesis, mesenchymal stem cells, AMPK

## Abstract

Thyroid hormone (TH), triiodothyronine (T3), and thyroxine (T4), which are released from the thyroid, control many cellular processes in various cell types. It is worth noting that TH plays a complex role in skeletal metabolic balance, and few studies have investigated whether TH exerts any effects on osteogenesis in bone mesenchymal stem cells (MSCs). We explored the effects of T3 on bone morphogenetic protein 9 (BMP9)-induced osteogenesis, which process is considered the most important in the osteogenic differentiation of C3H10T1/2 cells. *In vitro* osteogenesis was analyzed by alkaline phosphatase (ALP) activity and staining, bone mineralisation, and osteocalcin and osteopontin expression. Fetal limb explant cultures and ectopic MSC implantation further confirmed the role of T3. Finally, we examined the effect of AMPK/p38 signaling on the osteoblastic differentiation. T3 synergizes with BMP9 to enhance osteogenic marker expression induced by BMP9. Furthermore, T3 promotes BMP9-induced bone formation by fetal limb explant cultures and ectopic MSC implantation. Co-treatment with BMP9 and T3 can promote AMPK and p38 phosphorylation, and pretreatment with the AMPK inhibitor compound C and siRNA can abolish phosphorylation of p38 and BMP9+T3-induced ALP activity. Our results suggest that BMP9 and T3 promote osteogenic differentiation at least partially via the activation of the AMPK/p38 signaling pathway.

## Introduction

Bone is an important organ that provides support and protection for the whole body. Bone homeostasis is controlled by many types of bone cells from different lineages that are all derived from mesenchymal stem cells (MSCs), including osteoblasts, osteocytes, and chondrocytes. The commitment and differentiation of MSCs into osteogenic cells is closely related to the occurrence of bone metabolic diseases and is worthy of further study. MSCs commit to becoming osteoprogenitor cells and differentiate into pre-osteoblasts, which eventually become mature osteoblasts. This process is regulated by various signaling factors and hormones, including bone morphogenetic proteins (BMPs), Wnt, insulin-like growth factors (IGFs), fibroblast growth factors (FGFs), and Notch. Understanding the signaling pathways that govern osteogenic differentiation has significant implications for bone metabolic diseases.

Thyroid hormones (TH), triiodothyronine (T3), and thyroxine (T4) play complex roles in skeletal development. In adulthood, hypothyroidism and hyperthyroidism induce osteoporosis and osteoporotic fractures, which lead to a substantial economic burden for society. Bone tissue at the juvenile stage has high sensitivity to TH, and thyroid dysfunction leads to delays in bone formation and mineralization, stunted growth, or cretinism. On the other hand, childhood thyrotoxicosis accelerates bone formation, leading to craniosynostosis (Tsourdi et al., 2015). In terms of target cell types for TH action, studies showed that TH effects in various target cell types and much of them focus on chondrocytes, osteoblasts and osteoclasts, however, there is few studies investigate the effect of TH on MSCs, the source of chondrocytes and osteoblasts. Furthermore, TH has different mechanisms on various target bone cells by complex direct and indirect effects, involving many cytokines or growth factors, such as IGF-1, Wnt, PTHrP, and FGF. Thus the interactions and multiple possible points of interaction between them still need to be worked out (Kim and Mohan, 2013).

Bone morphogenetic proteins are key growth factors that have potent osteogenic capability, among which BMP9 has the most potent osteogenic activity *in vitro* and *in vivo*. BMP9 enhances osteoblast differentiation via the Smad 1/5/8-dependent and Smad1/5/8-independent pathways (Ren et al., 2016). Furthermore, a large number of factors and signaling pathways cross talk with BMPs and promote or restrict BMP9-induced osteogenic differentiation in various types of cells (Chen et al., 2016). And BMP9 has been the subject of few studies that have investigated the interaction between TH signaling and BMPs in the osteogenesis of MSCs (Nagayama et al., 2013; Zhang et al., 2016). Therefore, in this study, we aimed to analyze the effect of triiodothyronine on BMP9-induced osteogenic differentiation in C3H10T1/2 cells and to elucidate the underlying molecular mechanisms. The results showed that triiodothyronine enhanced BMP9-induced osteogenic differentiation, and the promotion of osteogenesis was partially mediated by the activation of AMPK/p38. And an increased mechanistic knowledge of TH on MSCs’ osteogenesis will remarkably accelerate the in-depth knowledge of fracture repair or other bone metabolic diseases therapeutics and development bone tissue engineering.

## Materials and Methods

### Cell Culture and Chemicals

The cell lines were cultured as previously described (Huang et al., 2012; Gao et al., 2013; Lindley et al., 2016). T3 (Sigma, United States) was dissolved in 1.0 N NaOH, and compound C (Skelleck, United States), and SB203580 (Skelleck, United States) were dissolved in DMSO.

### Construction of Recombinant Adenoviruses Expressing BMP9 and GFP

Recombinant adenoviruses were designed and produced with the AdEasy system as described previously (Aguilar et al., 2007; Gao et al., 2013; Lindley et al., 2016). The coding region of human BMP9 was PCR amplified and cloned into an adenoviral shuttle vector. Then, HEK293 cells were used to generate a recombinant adenoviruses that was ultimately designated as AdBMP9. AdBMP9 expresses BMP9 as well as GFP, while the analogous adenovirus expresses GFP only (AdGFP).

### Alkaline Phosphatase Activity and Staining

The cells were treated with AdGFP, AdBMP9, and/or T3 for different time and the Great Escape SEAP Chemiluminescence assay kit (BD Clontech, United States) was used to measure ALP activity in cells as previously described (Huang et al., 2012; Gao et al., 2013; Lindley et al., 2016). Total cellular protein levels was measured by BCA kit (Beyotime, China), and the results was the ratio of ALP activity and total cellular protein levels. After the cells were washed by PBS (GIBCO, United States) and fixed with 4% paraformaldehyde (Beyotime, China) on day 7 post-treatment, ALP staining was then performed using an ALP Staining Assay Kit (Beyotime, China).

### Alizarin Red Staining

Alizarin red staining was conducted as previously described (Aguilar et al., 2007; Gao et al., 2013; Lindley et al., 2016). C3H10T1/2 cells were seeded in 6-well cell culture plates and treated as previously described (Huang et al., 2012; Gao et al., 2013; Lindley et al., 2016). Then, at the chosen time point, 21 days post-treatment, the cells were washed and fixed. The samples were then incubated with 2% alizarin red (Sigma-Aldrich, United States) for 30 min. After washing with PBS, the mineralized nodules were observed through light microscopy (Leica DMI 3000B, Germany).

### Immunohistochemical Staining

Cells were permeabilized with 0.1% Triton-X (Sigma, United States) for 15 min at room temperature after fixed with 4% paraformaldehyde and washed with PBS as previously described on day 14 (Aguilar et al., 2007; Gao et al., 2013; Lindley et al., 2016). Then the cells were blocked in 5% BSA (Beyotime, China) for 60 min. Then, the cells were incubated with antibody (osteocalcin, sc30045, Santa Cruz Biotechnology; osteopontin, ab91655, Abcam) overnight at 4°C. The next day, the cells were washed and incubated with secondary antibody (Beyotime, China) according to the protocol. Before examined under a microscope, the cells were incubated with diaminobenzidine (DAB).

### Real-Time RT-PCR

Total RNA was extracted from cells treated as previously described at day 7 and then the RNA was reverse transcribed into cDNA (Takara, Japan). Quantitative real time PCR was conducted as previously described (Aguilar et al., 2007; Gao et al., 2013; Lindley et al., 2016). Primers are listed as follows: For *OPN* gene, the forward primer was 5′-AGCAAGAAACTCTTCCAAGCAA-3′, the reverse primer was 5′-GTGAGATTCGTCAGATTCATCCG-3′; For *OCN* gene, the forward primer was 5′-CTGACCTCACAGATCCCAAGC-3′, and the reverse primer was 5′-TGGTCTGATAGCTCGTCACAAG-3′; For *GAPDH* gene, the forward primer was 5′-AGGTCGGTGTGAACGGATTTG-3′, and the reverse primer was 5′-TGTAGACCATGTAGTTGAGGTCA-3′.

### Western Blotting Analysis

Western blotting analysis was performed according to the protocol. Protein samples were collected in RIPA lysis buffer (Beyotime, China), followed by sodium dodecyl sulfate-polyacrylamide gel electrophoresis (SDS-PAGE), then the separated proteins were transferred to polyvinylidene difluoride (PVDF) membranes (Millipore, United States). The membrane was blocked with blocking buffer (Beyotime, China) and incubated with antibodies against AMPK (2532, Cell Signaling), p-AMPK (2535, Cell Signaling), p38 (9212, Cell Signaling), p-p38 (9211, Cell Signaling), osteocalcin (sc30045, Santa Cruz Biotechnology), osteopontin (ab91655, Abcam), and GADPH (AG019, Beyotime) at 4°C overnight. Goat anti-mouse antibody and goat anti-rabbit antibody (A0216 and A0208, Beyotime) labeled with HRP were used as secondary antibodies for 1 h at room temperature.

### Transient Transfection With Small Interfering RNAs

C3H10T1/2 cells were plated in six-well plates and AMPKα1/2 siRNA (Aguilar et al., 2007) or control siRNA (GenePharma, China) was transfected into the cells with Lipofectamine 2000 transfection reagent (Invitrogen, United States) according to the manufacturer’s instructions. The sense strands of siRNAs for AMPKα was 5′-AAGAGAAGCAGAAGCACGACG-3′. And the sense strands control was 5′-AAGCCGGTATGCCGGT TAAGT-3′.

### Fetal Limb Explant Culture

Fetal limbs were prepared from mouse embryos as described previously (Aguilar et al., 2007; Gao et al., 2013; Lindley et al., 2016) and cultured in DMEM containing 0.5% BSA, ascorbic acid (50 mg/ml), β-glycerophosphate (1 mM), and 1% penicillin and streptomycin (Sigma, United States) for 12 days after treatment (*n* = 5 per group). At day 10, calcein (100 mM, Sigma, United States) was added to the medium to trace the new bone formation. Finally, the limbs were observed under a fluorescence microscope for histological evaluation.

### MSC Implantation and Micro-Computed Tomography Analysis

The subcutaneous implantation of MSCs and the induction of ectopic bone formation were performed as described previously (Aguilar et al., 2007; Gao et al., 2013; Lindley et al., 2016). Cells were treated with AdGFP or AdBMP9 alone or in combination with triiodothyronine for 7 days, and then the cells were collected for subcutaneous injection (5 × 10^6^ cells per injection) into male athymic nude mice (5 per group, 4–6 weeks old, and Shanghai Laboratory Animal Centre, China). Then, the mice were treated with triiodothyronine (0.1 μg/g/d) or PBS subcutaneously for 5 weeks. At the chosen time point, the animals were euthanized. The implantation sites were retrieved for scanning by micro-CT (μCT 80; Scanco Medical, Zurich, Switzerland) and for histological evaluation. All experiments were performed in accordance with the guidelines for animal experimentation of the Ethics Committee of Xinhua Hospital (Approval No. XHEC-F-2018-020). All the tissues were fixed with 4% paraformaldehyde and decalcified with 10% EDTA decalcifying solution, then embedded in paraffin. Haematoxylin and eosin (H&E) and Masson’s trichrome stain of serial sections were used for histological evaluation as previously described (Aguilar et al., 2007; Gao et al., 2013; Lindley et al., 2016).

### Statistical Analysis

All the results are expressed as the mean ± standard deviation (SD) of at least three independent experiments. Student’s *t*-test or one-way analysis was used for the variable comparisons, and a *P* value < 0.05 was considered statistically significant.

## Results

### Triiodothyronine Synergistically Promotes BMP9-Induced ALP Activity in MSCs

**FIGURE 1:**
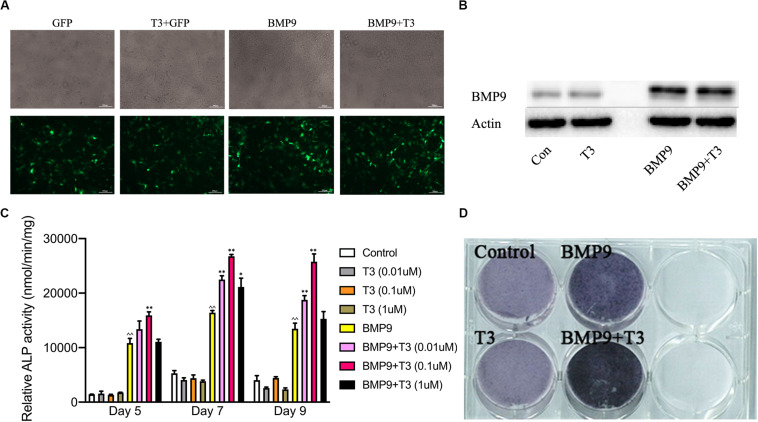
Triiodothyronine potentiates the BMP9-induced early osteogenic marker alkaline phosphatase. **(A)** Infection of C3H10T1/2 cells with AdGFP and AdBMP9. Subconfluent C3H10T1/2 cells were infected with AdBMP9 or AdGFP for 48 h. The GFP signal was observed under a fluorescence microscope. **(B)** Western blot results showed endogenous expression of BMP9 in C3H10T1/2 cells. **(C)** Triiodothyronine potentiates BMP9-induced ALP activity. Subconfluent C3H10T1/2 cells were infected with AdBMP9 or AdGFP in the presence or absence of triiodothyronine stimulation (at different concentrations). A quantitative ALP activity assay was conducted at the indicated time points. **(D)** C3H10T1/2 cells were fixed for an ALP histochemical staining assay at day 7. ^∧^^∧^*p* < 0.01 compared with the control group; *p < 0.05, and **p < 0.01 compared with the BMP9 group.

Many studies have shown previously that BMP9 is one of the most pro-osteogenic BMPs both *in vivo* and *in vitro*. T3 and TH have dual effects on the development of bone growth. To explore the influence of T3 on BMP9-induced MSCs’ osteogenic differentiation, we used a BMP9-expressing adenoviral vector that can effectively transduce C3H10T1/2 cells (Figures 1A,B). We then tested the osteoinductivity of BMP9 in C3H10T1/2 cells in the presence or absence of T3 stimulation. We found that BMP9 induced significant ALP activity and expression, while T3 alone did not induce any ALP activity. However, ALP activity was shown to increase in the presence of T3 at days 5, 7, and 9 when the concentration of T3 was 0.1 μM. Similar results were obtained from ALP histochemical staining assays at day 7 (Figures 1C,D).

### Triiodothyronine Potentiates the BMP9-Induced Osteogenic Markers Expression and Matrix Mineralization

**FIGURE 2:**
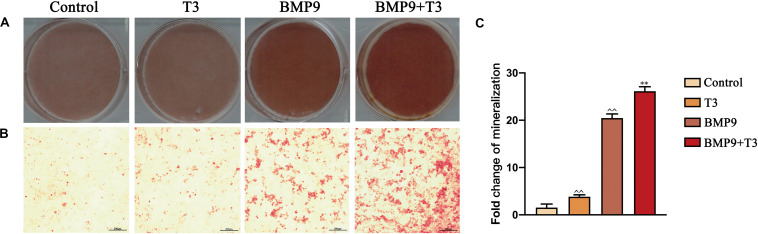
Triiodothyronine promotes BMP9-induced mineralization in C3H10T1/2 cells. **(A,B)** Macrographic and microscopic images (200X) of alizarin red S staining after 21 days of infection. **(C)** Statistical analysis of relative area (^∧^^∧^*P* < 0.01, compared with Control group; **P < 0.01, compared with BMP9 group).

**FIGURE 3:**
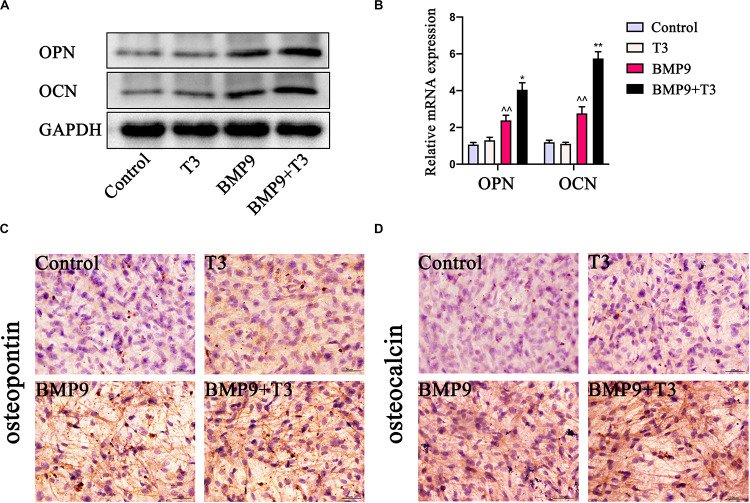
Triiodothyronine promotes BMP9-induced osteogenic marker expression in C3H10T1/2 cells. **(A,B)** Protein and mRNA expression of OCN and OPN induced by AdBMP9 for 9 days (^∧^^∧^*P* < 0.01, compared with Control group; **P < 0.01, compared with BMP9 group). **(C,D)** Immunohistochemical staining results showed the expression of OPN and OCN in C3H10T1/2 cells for fourteen days after infection (200X).

Furthermore, we also analyzed the late stage osteogenic differentiation of C3H10T1/2 cells treated with AdGFP or AdBMP9 alone or in combination with T3. Alizarin red S staining showed that T3 treatment increased BMP9-induced mineral nodule formation (Figure 2). Protein and mRNA analysis showed that T3 significantly increased osteocalcin and osteopontin expression when cells were infected with AdBMP9 (Figures 3A,B). Immunohistochemical (IHC) staining also confirmed the results (Figures 3D,E). Collectively, these results demonstrate that BMP9 and T3 synergistically enhance osteogenesis in MSCs.

### Triiodothyronine and BMP9 Act Synergistically to Promote Bone Formation in Mouse Embryo Limb Explant Cultures

**FIGURE 4:**
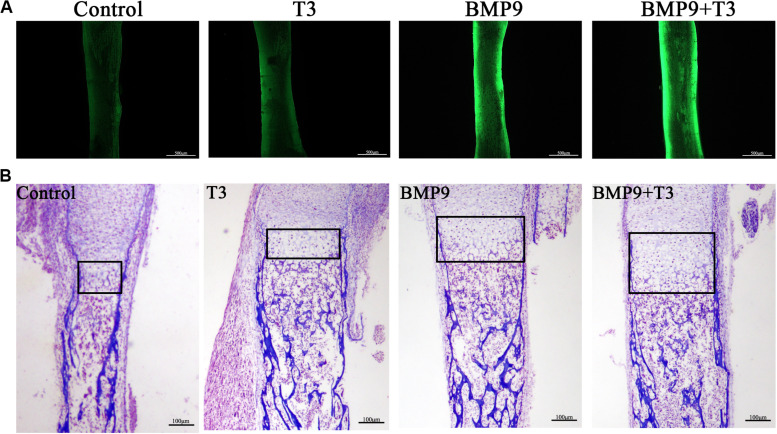
Triiodothyronine enhances the ability of BMP9 to expand the hypertrophic chondrocyte zone. **(A)** E18.5 mouse embryo limbs were isolated and cultured in DMEM supplemented with 0.5% BSA, 50 mg/ml ascorbic acid, 1 mM beta-glycerophosphate, and 100 mg/ml penicillin-streptomycin. The embryo limbs were infected with AdBMP9 or AdGFP in the presence or absence of triiodothyronine (0.1 μM) for 14 days. On day 12, calcein (100 mM) was added to the culture medium. The harvested limbs were subjected to fluorescence microscopy. **(B)** The harvested tissues were fixed, paraffin-embedded, and subjected to Masson’s trichrome staining. The boxed areas indicate the growth plate. Representative images are shown.

We sought to analyze the effect of T3 on developing bone by using fetal limb culture assays. E18.5 mouse embryo limbs were isolated (*n* = 5 each group) and infected with AdBMP9 and AdGFP in the presence or absence of T3 (0.1 μM). At the endpoint of each culture, the fluorescent dye calcein was used to show new bone formation. We found that treatment with BMP9+T3 induced new bone formation to the greatest extent compared with the control treatment (Figure 4A). Consistent with our previous reports, the histological evaluation revealed that BMP9+T3 treatment led to a significant expansion of the growth plate (Figure 4B).

### Enhancement of BMP9-Induced Ectopic Bone Formation by Triiodothyronine

**FIGURE 5:**
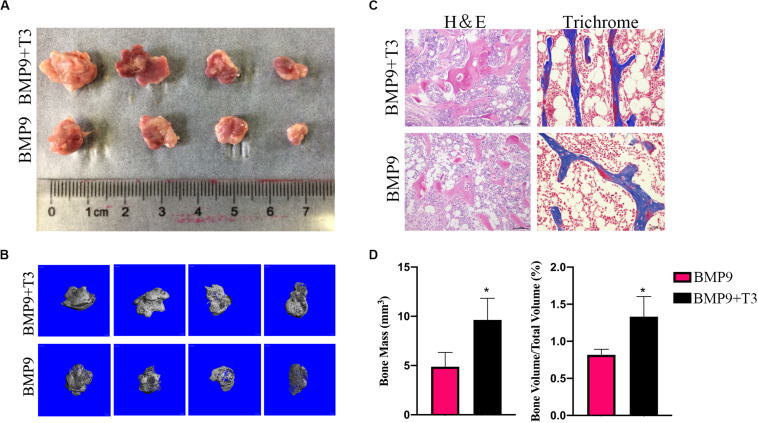
**(A)** Macrographic images of ectopic bones from the BMP9 and BMP9 + triiodothyronine groups after 5 weeks. Ectopic bones did not form in the control and triiodothyronine groups. **(B)** 3D reconstruction of bone masses by micro-CT analysis. The bone masses were evaluated and analyzed with the μCT 80 system. **(C)** H&E staining and Masson’s trichrome staining of serial sections of paraffin-embedded ectopic bones. **(D)** Analysis of bone mass and relative bone density. *p < 0.05.

We focus on ectopic bone formation ability to further investigate the osteogenic effect of a combination of BMP9 and T3. We pretreated C3H10T1/2 cells with AdBMP9 and/or T3, and then the collected cells were subcutaneously injected into male athymic nude mice, after which the mice were treated with T3, and/or PBS subcutaneously. After 5 weeks, all nude mice were sacrificed, and increases in bone mass were only detected in the groups treated with AdBMP9 alone and with T3 (Figure 5A). Micro-CT scans and histological results showed that C3H10T1/2 cells treated with AdBMP9+T3 formed slightly larger bone masses and showed more mineralization (Figures 5B–D) than cells in the BMP9 group. Altogether, these data confirmed that triiodothyronine can accelerate BMP9-induced bone formation.

### Triiodothyronine Enhances BMP9-Induced Osteogenic Differentiation Through an AMPK-Dependent Pathway

**FIGURE 6:**
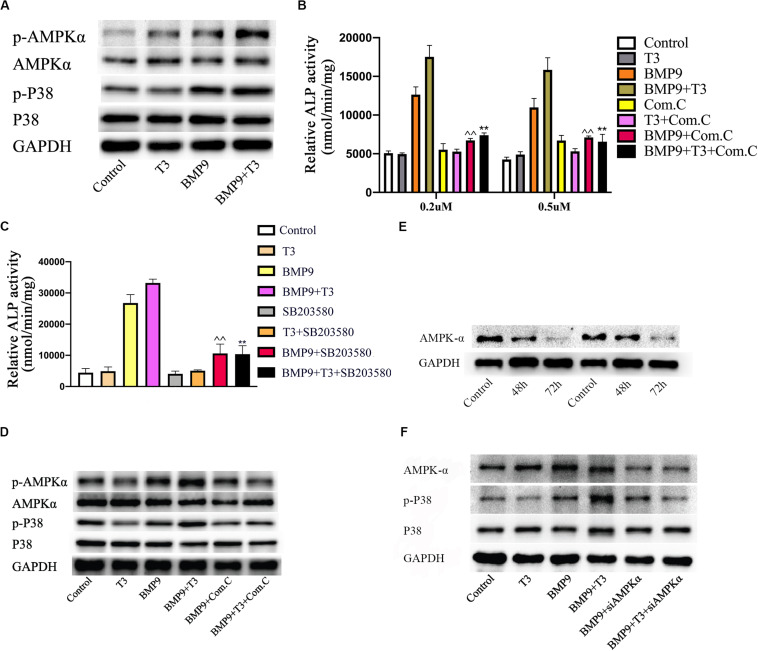
Triiodothyronine enhances BMP9-induced osteogenesis through the AMPK/p38 pathway in MSCs. **(A)** Cells were infected with AdGFP or AdBMP9 in the presence or absence of triiodothyronine for 72 h, and then protein levels were analyzed by Western blotting. **(B,C)** The effects of compound C and at different concentrations and SB203580 (5 μM) on ALP activity in all groups on day 7. ^∧^^∧^*p* < 0.01 compared with BMP9 group; **p < 0.01 compared with the BMP9+T3 group. **(D)** C3H10T1/2 cells were pre-treated with compound C and then treated with AdBMP9 or AdGFP in the presence or absence of triiodothyronine. Protein levels were analyzed by Western blotting. **(E)** Changes in protein levels 48 and 72 h after siAMPK treatment. **(F)** Treatment with siAMPK inhibited p38 phosphorylation.

Triiodothyronine has been found to induce osteocalcin synthesis in osteoblasts via the activation of AMPK or p38. Many studies have reported that AMPK and p38 activation can promote early during osteoblast differentiation and that their induction is required for normal bone formation *in vitro* and *in vivo*., p38 is involved in BMP9-induced osteogenic differentiation. Additionally, we examined the effects of T3 and BMP9 on AMPK and p38 MAPK activation. AMPK phosphorylation at Thr172 and p38 phosphorylation were significantly increased in the BMP9+T3 group, whereas the total protein levels remained unchanged (Figure 6A). However, the phosphorylation of AMPK and p38 was not affected by T3 in C3H10T1/2 cells. To further assess the significance of this change in osteoblast differentiation, inhibitors of AMPK and p38 were used. When C3H10T1/2 cells were treated with compound C, siRNA and SB203580, the ALP activity in the BMP9+T3 group was significantly inhibited (Figures 6B,C). Furthermore, exposure of C3H10T1/2 cells to compound C and siRNA (Figure 6D) inhibited the phosphorylation of p38 MAPK (Figures 6E,F). Collectively, these data indicate that the activation of AMPK and p38 MAPK mediate combined BMP9/T3-induced osteogenic differentiation and that AMPK may function as an upstream regulator of p38 MAPK.

## Discussion

We investigated whether T3 potentially has an effect on the promotion of BMP9-induced osteogenic differentiation of C3H10T1/2 cells, and we also examined the underlying molecular mechanisms. To clarify the effect, cells were treated with AdBMP9 alone or in combination with T3 and the expression of several osteogenic markers, such as ALP, OCN, and OPN were tested. Here, we found that ALP activity and OCN and OPN expression was most strongly induced in the co-treatment group. Furthermore, T3 was shown to promote BMP-9-induced ectopic bone formation in stem cell implantation assays and embryo limb explant culture assays. T3 and BMP9 co-treatment was shown to enhance the phosphorylation of AMPK and p38. In addition, both compound C, and siAMPK abolished the promotional effect, suggesting that BMP-9 may engage in crosstalk with T3 through the AMPK signaling pathway during osteogenic differentiation in MSCs. Collectively, our results showed that T3 has a potentially effect on the promotion of BMP9-induced osteogenesis and bone formation partially by activating AMPK/p38 signaling pathway.

Thyroid hormone has received considerable attention as a regulator of bone metabolism based on its role in stimulating the expression of osteocalcin, type 1 collagen, and ALP to enhance osteoblast activity and influence the activity and formation of osteoclasts through the increased expression of receptor activator of nuclear factor κB ligand and other cytokines involved in osteoclastogenesis (Tsourdi et al., 2015). In the process of promoting osteogenesis, TH regulates a number of osteogenic-related growth factor signaling factors, including IGFs, parathyroid hormone-related protein, FGFs, and Wnt, to influence skeletal growth (Kim and Mohan, 2013). TH also has also been shown to regulate energy metabolism involving cholesterol levels, lipolysis and gluconeogenesis via activating AMPK signaling pathway, which is also important for bone metabolism (Wojcicka et al., 2013; Mullur et al., 2014). BMP9, a member of the BMP family, is known to be the most potent BMP family osteogenic factor in MSCs. BMP9 also regulates several biological processes, such as glucose and lipid metabolism (Chen et al., 2003; Caperuto et al., 2008; Luo et al., 2017).

The potential of T3 to interactive with BMP signaling pathway has been supported by several studies. T3 has been shown to function cooperatively with BMP4 to regulate cartilage differentiation and endochondral bone formation. In the processes of chondrocyte maturation and the synthesis of collagen X, T3 treatment stimulated the expression of BMP 4, which was accompanied by the downregulated expression of the BMP inhibitor Noggin (Lassova et al., 2009). Another study also showed that T3 signaling is essential for BMP4-induced colorectal cancer cell differentiation (Catalano et al., 2016). However, we found that T3 didn’t influence the mRNA level of BMP receptor (Supplementary Figure S2 and Supplementary Table S1). Furthermore, the result of western blot showed that T3 treatment can slightly promote phosphorylation level of Smad1/5/8, while when co-treated with BMP9 the phosphorylation of p-Smad1/5/8 was also slightly increased (Supplementary Figure S3). In addition, to the best of our knowledge, several studies have demonstrated that TH is a major regulator of IGF-1 expression, β-catenin accumulation, and TCF/LEF transcriptional activity (O’Shea et al., 2005; Xing et al., 2012). IGF signaling is critical for the development and homeostasis of bone (Chen et al., 2010a, 2016). Both IGF1 and IGF2, two ligands involved in IGF signaling, have been crosstalk with BMP9 in osteogenic differentiation of MSCs evidenced by the activation of Smad signaling (Chen et al., 2010b; Li et al., 2015). Wnt/β-catenin signaling pathway is a classical osteogenic differentiation pathway and have synergistically osteogeneic effect when treated with BMP9 simultaneously (O’Shea et al., 2005). Likewise, our results suggest that T3 alone has no significant effect on ALP activity, OPN expression. In C3H10T1/2 cells infected with AdBMP9, we found that T3 enhanced BMP9-induced osteogenic marker expression, matrix mineralization, new bone formation and increased the phosphorylation of AMPK/p38. Thus, our data suggested that the augmentation of BMP9-induced osteogenesis by T3 may be partly mediated by AMPK/p38 signaling.

The effects of THs are mediated through thyroid hormone receptors (TR) that act as ligand dependent transcription factors, but we found that the mRNA expression of both TRα and TRβ showed no significant changes in our study (Supplementary Figure S4 and Supplementary Table S1). Futrther, several studies reported that TH can stimulate AMPK signaling pathway through rapid, transcription-independent (non-genomic) effects in other cells and the activation of AMPK is required for osteoblast differentiation in both MC3T3E1 cells and primary murine osteoblasts (Shah et al., 2010; Xi et al., 2016; Wang et al., 2018). It is reported that triiodothyronine can induce osteocalcin synthesis in MC3T3E1 cells via the activation of AMPK or p38 (Ishisaki et al., 2004; Chen et al., 2010b; Kondo et al., 2013). In addition, T3 induced IGF-1, and IGFBP-2 expression can stimulate AMPK activation during MC3T3E1 cells osteogenesis (Xing et al., 2012; Xi et al., 2016). Our results significantly support these observations by confirming that T3 and BMP9 treatment stimulate osteogenic differentiation via the phosphorylation of AMPK. Nevertheless, we did not find that T3 can induce osteocalcin expression in C3H10T1/2 cells or induce the phosphorylation of AMPK and p38 MAPK. It is conceivable that the role of T3 may be different in different cells and cell stages. It was reported that p38 may function as a downstream signaling molecule of AMPK in several studies. AMPK was reported to interact with p38 to regulate glucose metabolism, BMP2 expression, and COX-2 expression, all of which play an important role in the processes of bone formation and remodeling (Xi et al., 2001; Hou et al., 2008; Huang et al., 2010). Additionally, COX-2 was reported to form an important regulatory loop with BMP9 and to induce osteogenic differentiation in MSCs (Wang et al., 2013). Additionally, p38 is involved in BMP9-induced osteogenic differentiation (Xu et al., 2012; Kamel et al., 2017). Then, we examined the potential role of p38 on the effects of T3 on BMP9-induced osteogenesis. Pretreatment of C3H10T1/2 cells for 60 min with SB203580 markedly attenuated ALP activity. In addition, the phosphorylation of p38 were inhibited by compound C and AMPK siRNA in our study. Therefore, we proposed that the mode of action of T3 and BMP9, which leads to the induction of osteogenesis, is partly achieved by the sequential activation of AMPK and p38 MAPK. However, several studies have also reported that the activation of p38 is not influenced by the modulation of AMPK in the control of myocardial glucose metabolism and tumourigenesis. Thus, further studies are required to clarify the mechanism involved in the AMPK/p38 pathway in MSC osteogenic differentiation.

Overall, we found that the synergistical effects of T3 on BMP9-induced MSCs osteogenesis may be mediated by increased AMPK/p38 signaling. These findings provide insights into the complex effect of T3 involved in osteogenic differentiation in MSCs.

## Data Availability Statement

The raw data supporting the conclusions of this article will be made available by the authors, without undue reservation.

## Ethics Statement

The animal study was reviewed and approved by Animal Ethical and Welfare Committee of Xin Hua Hospital Affiliated to Shanghai Jiao Tong University School of Medicine (Approval No. XHEC-F-2018-020).

## Author Contributions

XC and YG designed the study. XC, TJ, CX, and YH performed the experiments. XC wrote the original draft of the manuscript. YG reviewed and edited the manuscript. All authors contributed to the article and approved the submitted version.

## Conflict of Interest

The authors declare that the research was conducted in the absence of any commercial or financial relationships that could be construed as a potential conflict of interest.
