# Supplementary material for: Triiodothyronine Potentiates BMP9-Induced Osteogenesis in Mesenchymal Stem Cells Through the Activation of AMPK/p38 Signaling
Source: Front Cell Dev Biol. 2020 Jul 31;8:725. doi: 10.3389/fcell.2020.00725 (PMC7413205; doi:10.3389/fcell.2020.00725)
Supplement: Supplementary file 1 [file Table_1.DOCX]

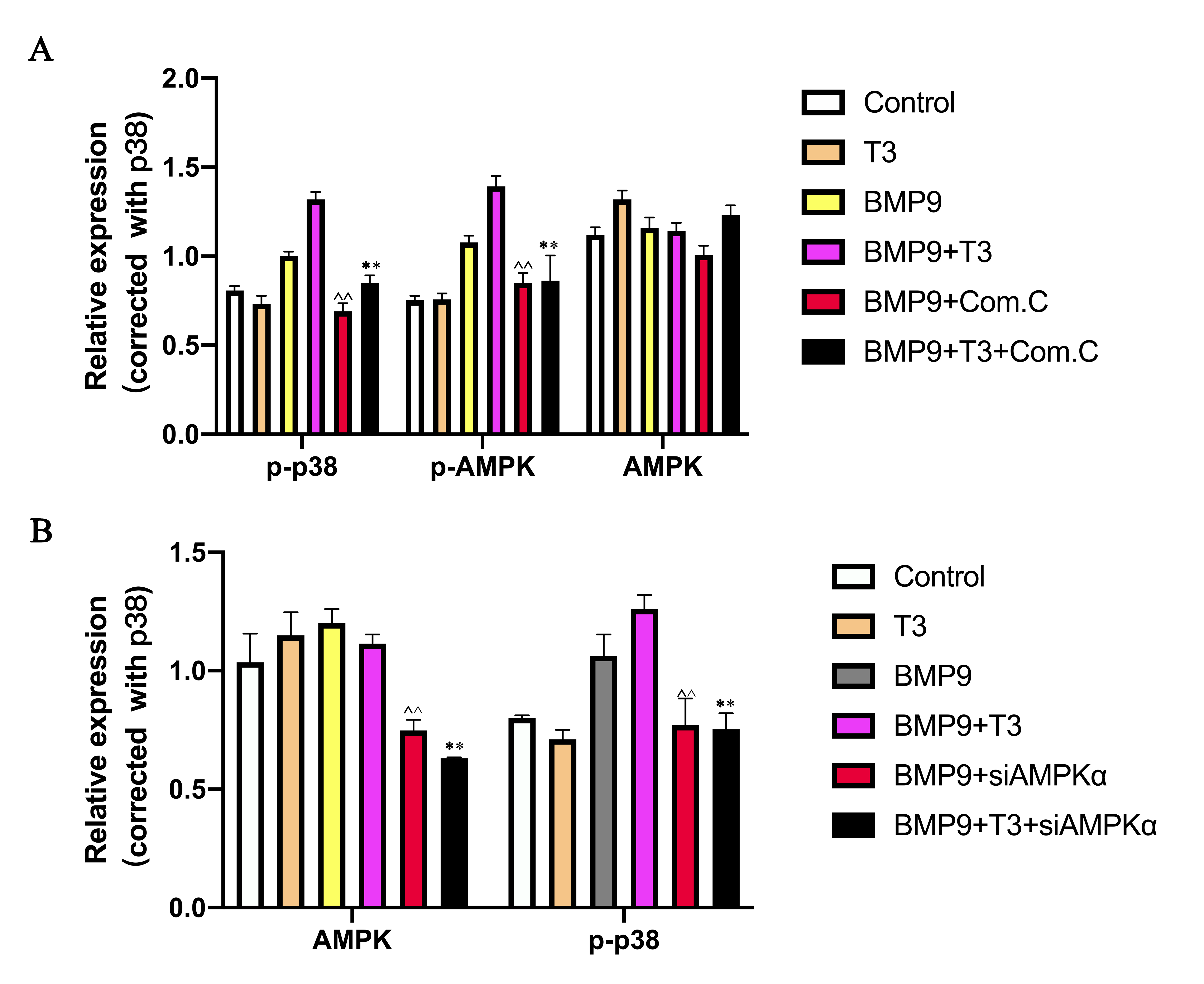


Figure S1. Relative expression of p-p38, p-AMPK and AMPK corrected with MAPK p38.


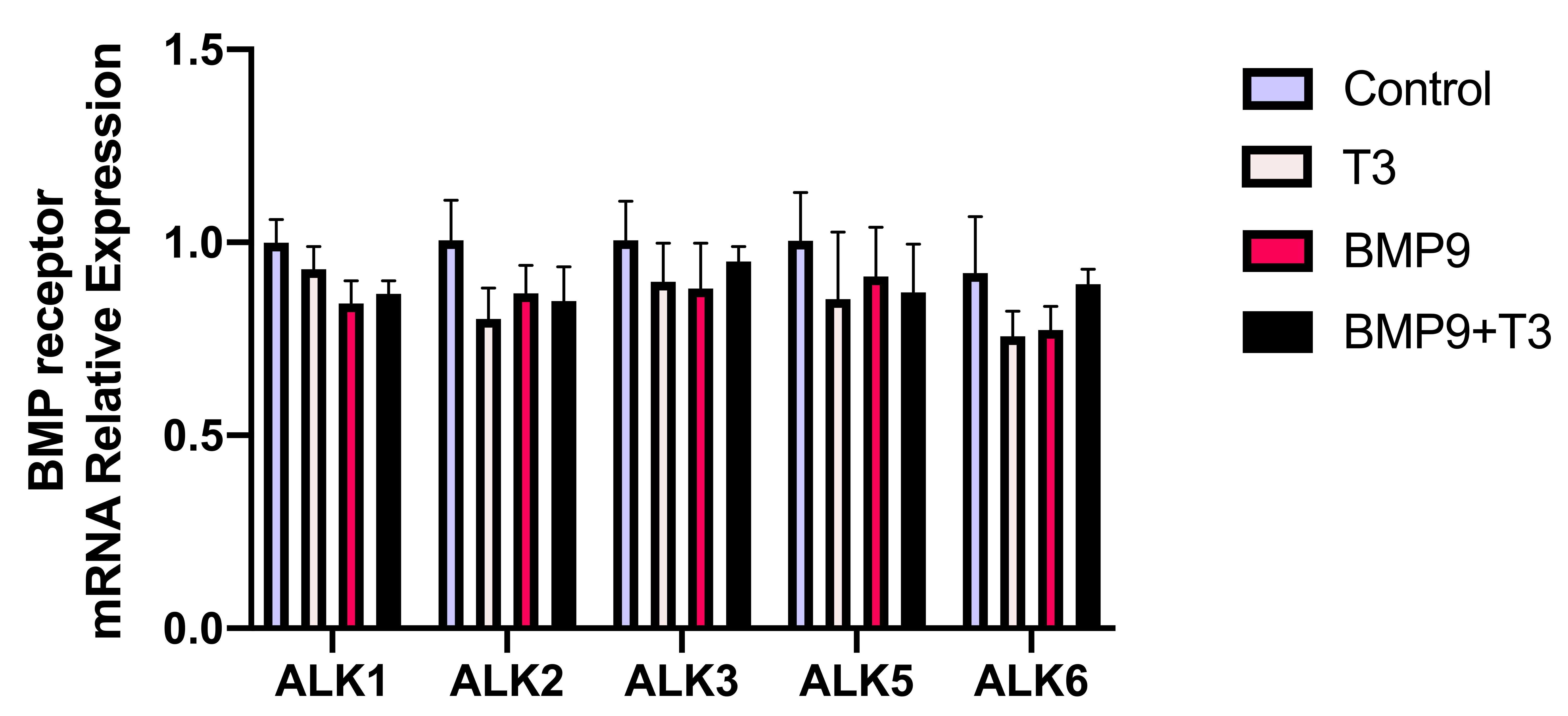


Figure S2. mRNA expression of ALK1, ALK2, ALK3, ALK5 and ALK6 induced by AdBMP9 and T3 treatment after 3 days.


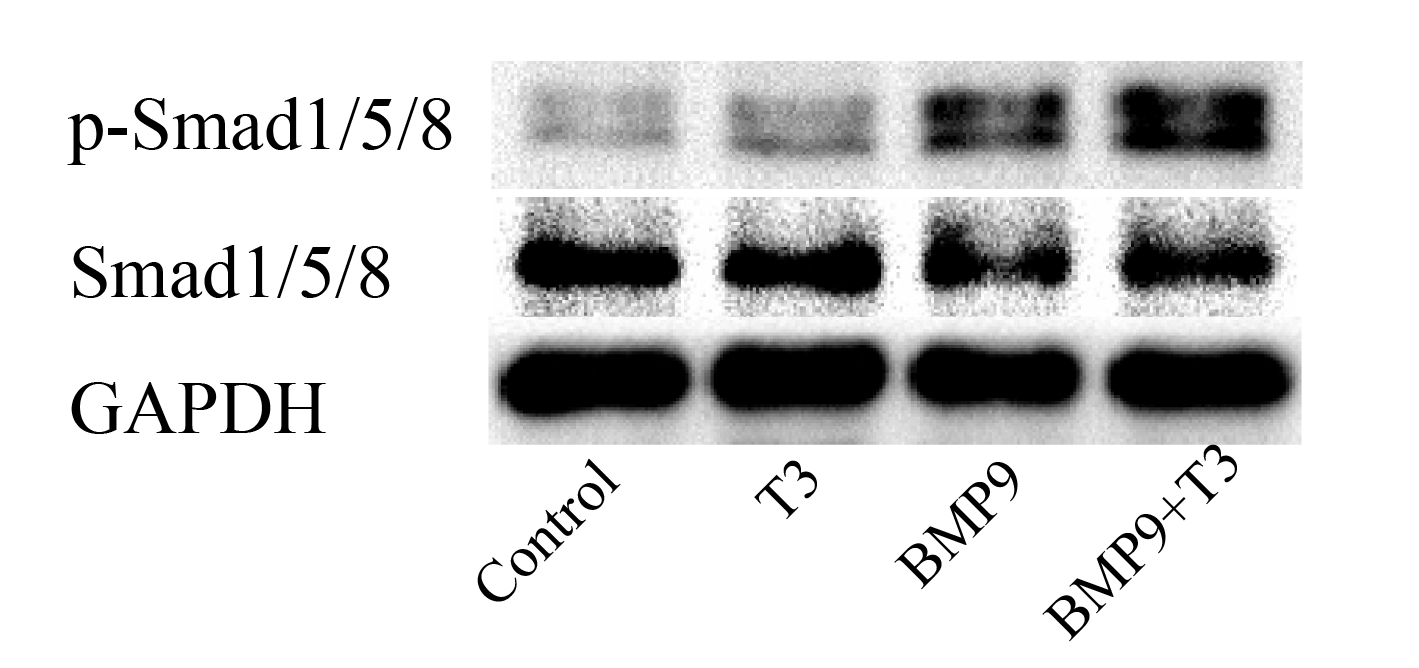


Figure S3. Protein levels of Smad1/5/8 and p-Smad1/5/8 were analysed by western blotting


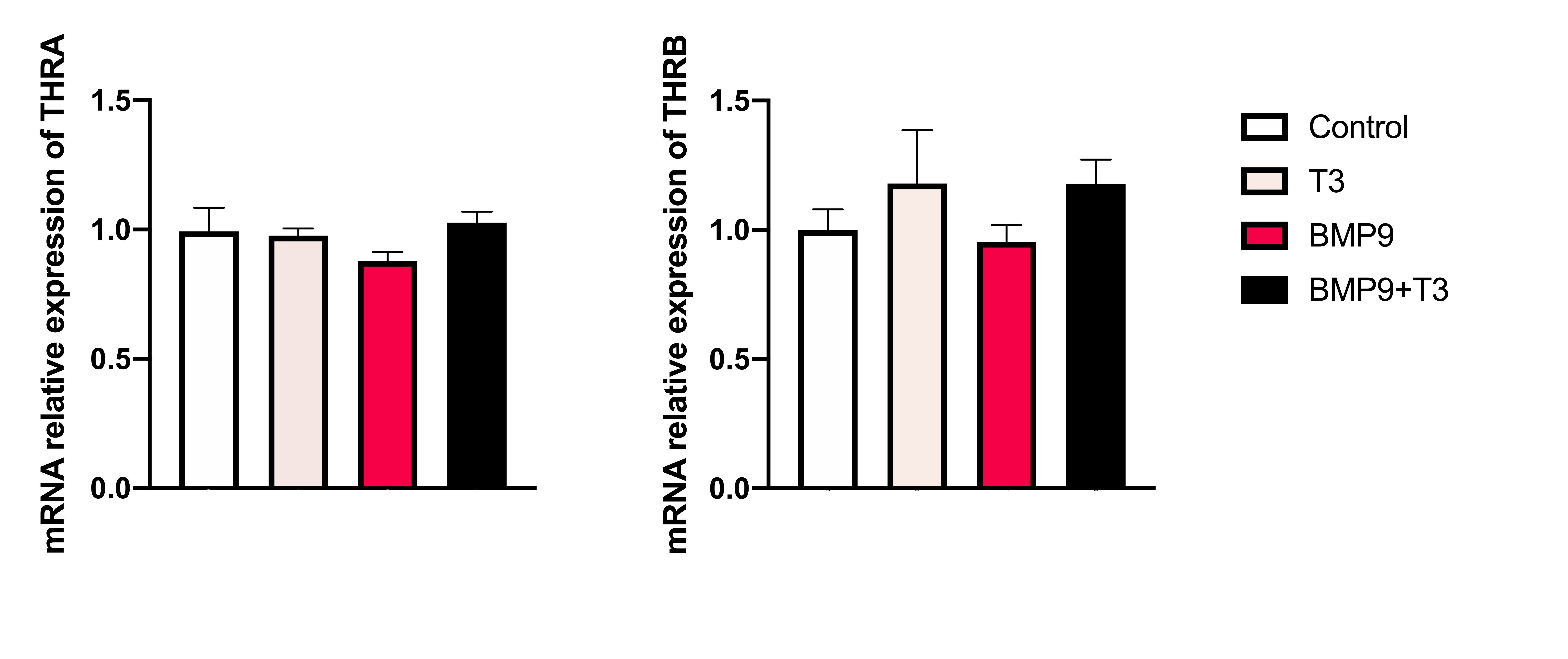


Figure S4. mRNA expression of THRA and THRB induced by AdBMP9 and T3 (0.1uM) after 3 days treatment.

Table S1. Sequence of the primers used in the qPCR analysis of mRNA expression.

| Gene | The forward primer | The reverse primer |
| --- | --- | --- |
| ALK1 | GGCGATGAAGCCTAGGATGTT | GGCGATGAAGCCTAGGATGTT |
| ALK2 | TGGTGAGCAATGGTATAGTGGA | CCACACAGACAACTTTCCTCAT |
| ALK3 | ATGCAAGGATTCACCGAAAGC | AACAACAGGGGGCAGTGTAG |
| ALK5 | TTGCAGACTTGGGACTTGCT | GGGCCATGTACCTTTTAGTGC |
| ALK6 | CACCACTGTCCGGAAGACTC | CCAGAGGTGACAACAGGCAT |
| THRA | CTGACCTCCGCATGATCGG | GGTGGGGCACTCGACTTTC |
| THRB | CCAGAGGTACACGAAGTGTGC | AGGTTTCCAGGGTAACTACAGG |
| GAPDH | AGGTCGGTGTGAACGGATTTG | TGTAGACCATGTAGTTGAGGTCA |
